# The Pupillary Orienting Response Predicts Adaptive Behavioral Adjustment after Errors

**DOI:** 10.1371/journal.pone.0151763

**Published:** 2016-03-24

**Authors:** Peter R. Murphy, Marianne L. van Moort, Sander Nieuwenhuis

**Affiliations:** 1 Institute of Psychology and Leiden Institute for Brain and Cognition, Leiden University, Leiden, The Netherlands; 2 Department of Educational Science, Leiden University, Leiden, The Netherlands; Ghent University, BELGIUM

## Abstract

Reaction time (RT) is commonly observed to slow down after an error. This post-error slowing (PES) has been thought to arise from the strategic adoption of a more cautious response mode following deployment of cognitive control. Recently, an alternative account has suggested that PES results from interference due to an error-evoked orienting response. We investigated whether error-related orienting may in fact be a pre-cursor to adaptive post-error behavioral adjustment when the orienting response resolves before subsequent trial onset. We measured pupil dilation, a prototypical measure of autonomic orienting, during performance of a choice RT task with long inter-stimulus intervals, and found that the trial-by-trial magnitude of the error-evoked pupil response positively predicted both PES magnitude and the likelihood that the following response would be correct. These combined findings suggest that the magnitude of the error-related orienting response predicts an adaptive change of response strategy following errors, and thereby promote a reconciliation of the orienting and adaptive control accounts of PES.

## Introduction

A robust finding across psychological tasks that measure reaction time (RT) is that RTs slow down after an error is committed [1,2]. This post-error slowing (PES) has often been viewed as a product of the adaptive recruitment of cognitive control following error commission [3–6]. According to this account, an error signals the need for increased control, which in turn leads to more cautious decision-making and the prevention of additional errors. Recently, however, an alternative explanation of PES has emphasized that the very process of registering a salient, unexpected event like an error can temporarily interfere with subsequent information processing. Thus an error-related ‘orienting response’ (OR), which manifests in the transient activation of central and peripheral physiological systems related to alertness and the re-orientation of attention [7–9], might lead to RT slowing by disrupting task performance on the following trial [10–13]. The adaptive control and orienting accounts of PES can be distinguished by their predictions regarding the effect of error commission on the accuracy of subsequent responding: The former account specifies that the implementation of control should yield better-than-average accuracy after an error, whereas the latter specifies that orienting-related interference will lead to worse-than-average accuracy.

Adaptive control and interference by orienting have been portrayed as competing, mutually exclusive mechanisms of PES [10,14]. However, consideration of how the length of the interval between error commission and subsequent trial onset (response-to-stimulus interval; RSI) affects post-error behavior suggests that these accounts may be reconcilable. Evidence for PES accompanied by post-error decreases in accuracy has typically come from studies that employed relatively short (<~800ms) RSIs [11,15,16]. At longer RSIs, by contrast, PES is smaller but still reliably observed, and post-error accuracy is either improved or unchanged relative to average accuracy levels [1,4,15–18]. This pattern of findings suggests that error-related orienting effects on post-error behavior may be contingent on the pace of the task being performed: If RSI is short and the OR is still developing when the subsequent stimulus is presented, post-error performance will be impaired; but, if RSI is long and the OR resolves before the next trial, orienting will not be detrimental and may even be a precursor to the implementation of adaptive control. Indeed, a *positive* effect of orienting on post-error behavior would be consistent with the long-recognized role of the OR in translating salient or unexpected information into attentional engagement and behavioral adjustment [19,20].

In the present study, we hypothesized that the error-related OR is a pre-cursor to adaptive behavioral adjustment when it is afforded sufficient time to resolve before subsequent trial onset. Specifically, we conjectured that the magnitude of error-related orienting would positively predict both PES and the likelihood of accurate post-error performance in such circumstances. We tested this novel hypothesis by asking participants to perform an auditory choice RT task with long (6 seconds) inter-stimulus intervals and measuring evoked pupil dilation. Pupil dilation exhibits long-known sensitivity to stimulus characteristics including prior probability [21,22], surprise [23–25], intensity [26] and motivational significance [27,28], which collectively establishes pupil dilation as one of a host of peripheral measures of the OR [29] (but see [30]). Moreover, evoked pupil dilation is typically observed to be significantly larger following erroneous compared to correct task responses [7,31,32]. We found that such error-related pupil dilation was positively related, at the single-trial level, to both RT slowing and the likelihood of correct responding on directly following trials, thereby promoting a reconciliation of the orienting and adaptive control accounts of PES.

## Materials and Methods

### Participants

Twenty-four individuals participated in the study for course credit or €7.50. Four participants were excluded from all analyses: three with <18 error trials after pupillometric artifact rejection and one with no variance in post-error accuracy. Thus we analyzed a final sample of 20 participants (18 female; mean age = 22.5 years, SD = 5.9). This pre-determined sample size is consistent with previous studies from our lab that investigated the neuro-cognitive basis of PES [13]. Participants provided written informed consent prior to testing, and all procedures were approved by the Leiden University Institute of Psychology ethics committee and conducted in accordance with the Declaration of Helsinki.

### Task Design

**Fig 1:**
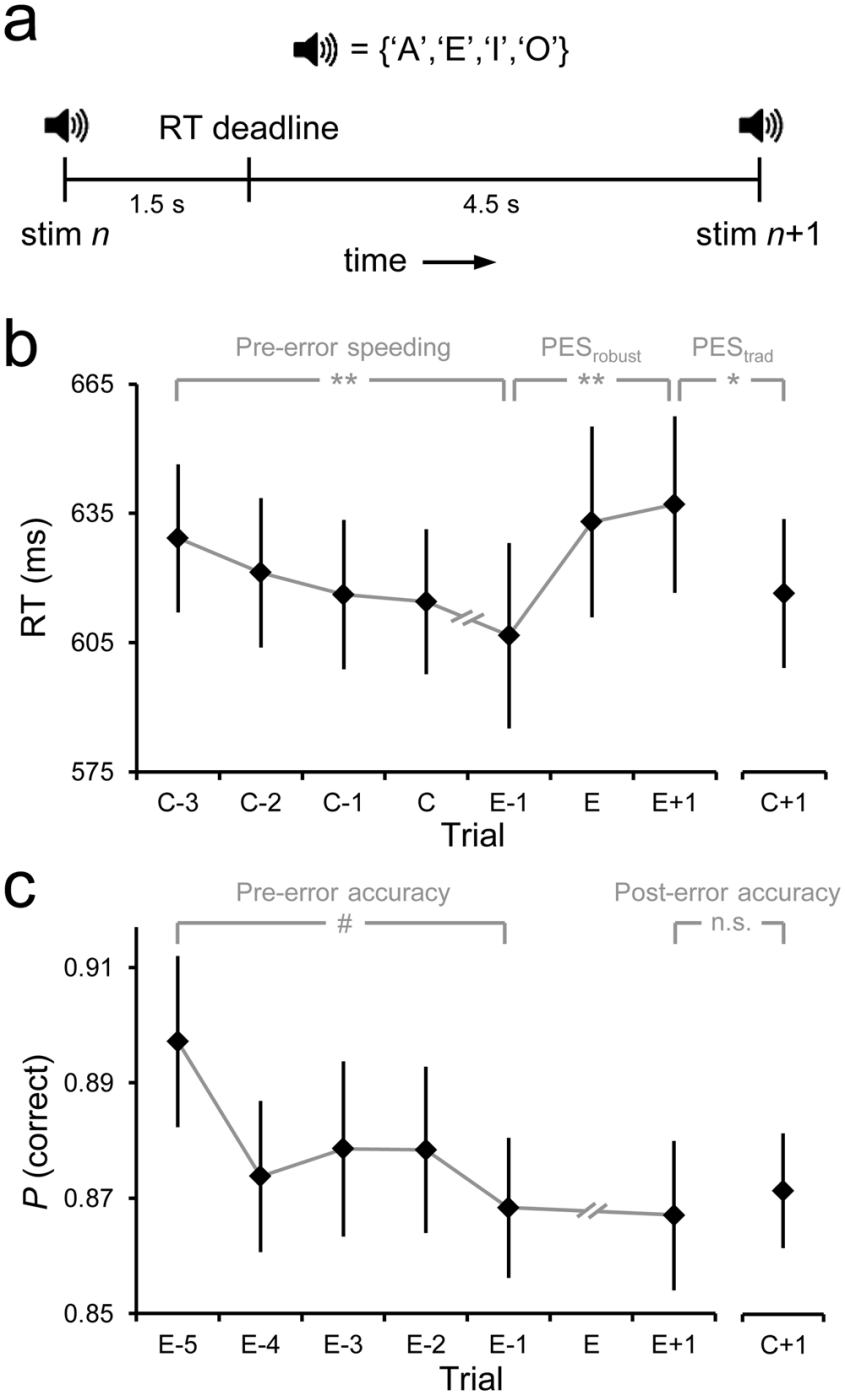
Task and behavioral results. (A) Timing of a trial of the auditory four-choice RT task. (B) Peri-error RTs, illustrating both pre-error speeding and PES. The latter was quantified using both ‘traditional’ and ‘robust’ methods (see Materials and Methods). (C) Peri-error response accuracy. Pre-error effects in *b* and *c* are linear contrasts across consecutive trials. Error bars indicate s.e.m. **p<0.01, *p<0.05, ^#^*p*<0.1.

We employed an auditory four-choice reaction time (RT) task (Fig 1A). On each trial participants were presented with one of four equiprobable, randomly ordered letters (A,E,I,O). The task was to respond to each letter with a finger press: left middle, left index, right index, and right middle for A-E-I-O, respectively.

Stimuli were delivered using E-Prime. Participants sat in a testing booth with moderate ambient luminance (21.8 cd/m^2^ with luminance meter in middle of booth, pointed at task display), 60cm from a monitor that displayed a central black fixation cross on a light-grey background (123.5 cd/m^2^). Auditory stimuli were presented binaurally via headphones for 300ms, and participants responded using the horizontally-aligned 1, 2, 8 and 9 keys on a keyboard for the A-E-I-O letters, respectively. The inter-stimulus interval was 6 seconds—pilot testing revealed that this interval length was sufficiently long for evoked pupil responses to return to baseline before subsequent trial onset. Participants were instructed to maintain fixation on the cross, minimize blinking and movement, and respond to stimuli as quickly as possible within a deadline of 1,500ms.

Participants first completed 20 practice trials accompanied by immediate post-response visual feedback indicating whether each response was ‘correct’, ‘incorrect’, or ‘too slow’. They were then administered at least 4 blocks of 100 trials without post-response feedback. For four participants, time constraints allowed a fifth block to be administered. Short breaks were given between blocks, during which participants received feedback about performance during the preceding block. To increase the number of error trials, we asked participants to increase their response speed if their average accuracy was above 90%.

### Pupillometric Recording and Preprocessing

Binocular pupil diameter was recorded at 60Hz using a Tobii T120 eye-tracker. The eye-tracker was calibrated to each participant prior to testing using Tobii Studio software. All offline analyses were carried out in Matlab. Left and right pupil diameters were averaged and eye-blinks and other noise transients were removed using a linear-interpolation algorithm that restricted interpolation to periods of consecutive data loss shorter than 1 second. Pupillometric time series were then segmented into epochs from -1 to +3s relative to stimulus onset. Remaining artifacts were identified by applying amplitude (any sample <1mm) and gradient (any difference in consecutive samples >0.1mm) criteria to the segmented data. In the final sample of participants, 6.0±8.9% of trials contained at least one artifactual sample and these trials were discarded from pupillometric analyses. Pupillometric data for the remaining trials were low-pass filtered to 5 Hz (fourth order Butterworth)–the pupillary dynamics of interest presently are considerably slower than this frequency cutoff and filtering served to remove higher-frequency noise from single-trial measurements.

### Behavioral Analysis

PES was quantified for each subject in two complementary ways. First we employed the ‘robust’ approach [33] that compares mean RT on trials following errors to mean RT on trials directly preceding errors. Only errors that were both preceded and followed by correct trials are included in this comparison. This ‘PES_robust_’ metric is unaffected by fluctuations in global performance where periods of low accuracy are accompanied by generally slow RTs. However, PES_robust_ may be inflated by pre-error *speeding*. Thus, we also derived PES estimates by subtracting mean post-error RT from mean post-correct RT. This ‘PES_traditional_’ metric is potentially affected by global performance fluctuations but not by pre-error speeding.

We also examined the behavioral data for pre-error trends in response accuracy and RT. We calculated the mean accuracy on each of the five trials directly preceding errors and tested whether accuracy changed linearly with trial position via linear contrast. For RT, the analysis was restricted to correct pre-error trials. Errors were rarely preceded by sequences of five correct trials. To boost trial counts, we isolated any sequence of four correct trials that did not feature a pre-error trial, calculated mean RT for each trial position across these sequences, and concatenated this average sequence with the mean RT across correct trials that directly preceded errors. We then tested whether RTs in the concatenated sequence changed linearly with trial position via linear contrast.

### Pupil Dilation Analysis

**Fig 2:**
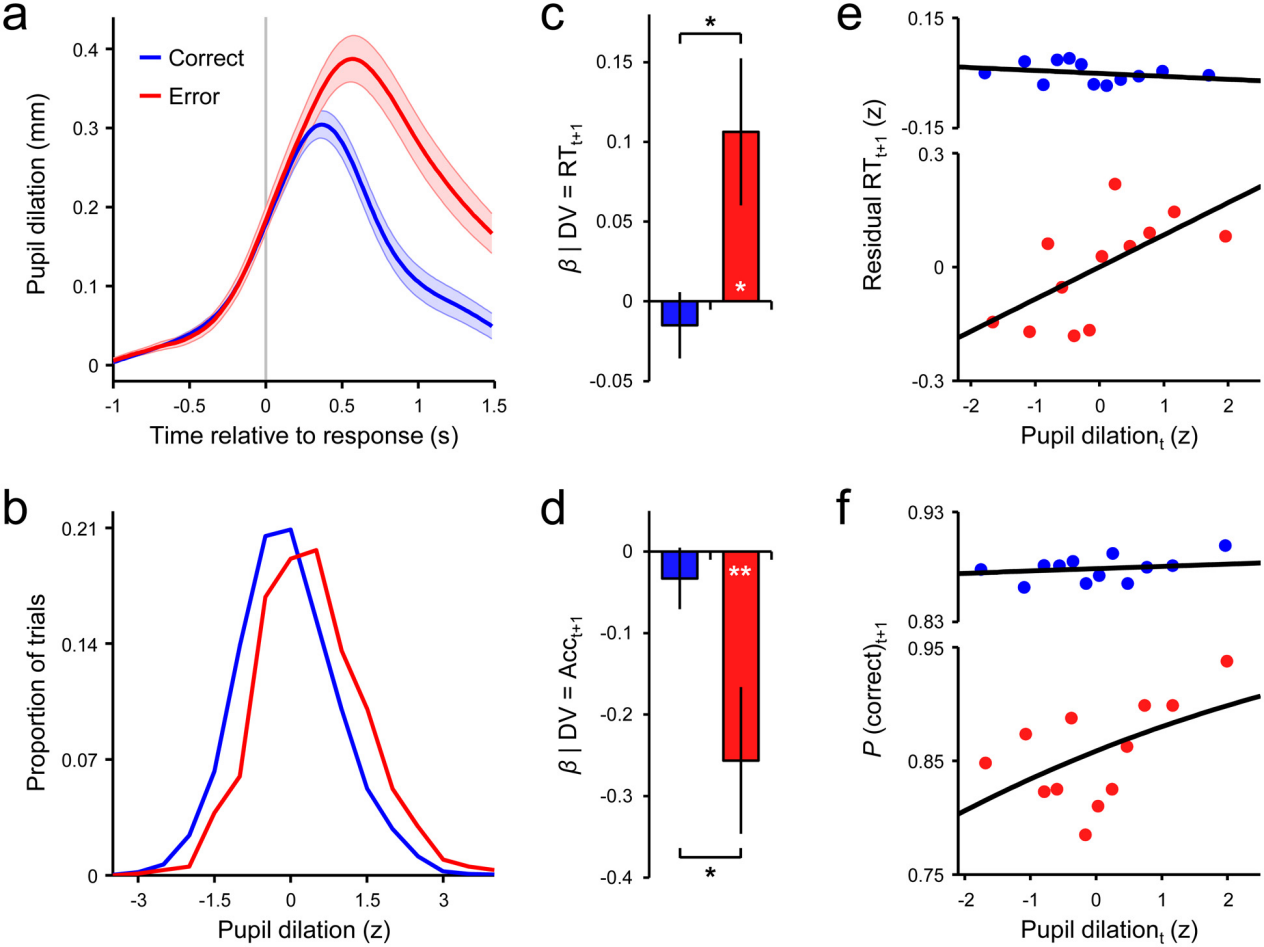
Relationships between pupil dilation and next-trial behavior. (A) Grand-average evoked pupil dilations conditioned on response accuracy (correct, error) and locked to response execution. (B) Distributions of single-trial pupil dilation amplitudes conditioned on response accuracy. Single-trial values were *z*-scored within subjects but across accuracy conditions, and pooled across subjects prior to plotting. Pooled distributions were normalized within condition such that each distribution integrates to 1. (C) Mean *β* coefficients from within-subjects linear regressions of next-trial RT onto current-trial pupil dilation for each accuracy condition (Eq 2, Materials and Methods). Significant effect of condition is derived from the interaction term of multi-factorial regression models that included pupil dilation and current-trial accuracy as predictors (Eq 1, Materials and Methods). (D) Mean *β* coefficients from within-subjects logistic regressions of next-trial accuracy on current-trial pupil dilation (Eqs 3 and 4, Materials and Methods). (E) Scatterplots illustrating the linear relationships between current-trial pupil dilation and next-trial RT, separately for correct and error trials. Data were *z*-scored within condition and within subjects, pooled across subjects, sorted by pupil dilation and grouped into 12 quantiles. Previous-trial RT was partialled out of next-trial RT prior to pooling (see Materials and Methods). (F) Scatterplots illustrating the logistic relationships between current-trial pupil dilation and next-trial accuracy, separately for correct and error trials. Shaded regions in *a* and error bars in *c* and *d* indicate s.e.m. **p = 0.01, *p<0.05.

Single-trial evoked pupil dilation was measured as the peak pupil diameter in the 0 to 1.5s following response execution, relative to a 1s pre-stimulus baseline. This post-response measurement reflected our focus on the response-related component of the pupillary OR, although reliable effects of the same nature as those reported below were also observed when a stimulus-aligned measurement window (0.5–2.5s) was employed. We interrogated relationships between pupil dilation on trial *t* and behavior on trial *t*+1 via single-trial within-subjects regression. Both correct and error trials were first considered in the same multiple regression models, and observed interactions were then decomposed using simpler models specific to each level of current-trial accuracy. To examine the relationship between pupil dilation and RT on the following trial, we fit the following linear regression model,

$$RT_{t+1}=\beta_{0}+\beta_{1}*Acc_{t}+\beta_{2}*Pupil_{t}+\beta_{3}*(Acc_{t}*Pupil_{t})+\beta_{4}*RT_{t-1}$$ (1)

where *RT*_t+1_ represents log-transformed, *z*-scored next-trial RT, *Acc*_t_ indicates current-trial accuracy (0 = correct, 1 = error), *Pupil*_t_ indicates *z*-scored current-trial pupil dilation, and *Acc*_t_*Pupil_t_ represents the interaction between *Acc*_t_ and *Pupil*_t_. The final term, representing log-transformed, *z*-scored RT on the preceding trial, controls for global fluctuations in RT that are not related to sequential behavioral adjustments much like PES_robust_ controls for such effects at the trial-averaged level. Although we do not report the results here, the selective relationship between error-related pupil dilation and next-trial RT (Fig 2C and 2E) was not dependent on the inclusion of this covariate. Only those instances where responses on trials *t*-1 and *t*+1 were both correct were included when fitting Eq 1. For all models, *z*-scoring of variables was carried out on a within-subject basis (i.e. using subject-specific across-trial mean and s.d. values), and *β*_i_ are per-subject fitted regression coefficients. To decompose the interaction effect, we fit the following models,

$$RT_{t+1}=\beta_{0}+\beta_{1}*Pupil_{t}+\beta_{2}*RT_{t-1}$$ (2)

separately to cases in which the response on trial *t* was correct or incorrect.

The relationship between pupil dilation and next-trial accuracy was examined via logistic regression. Again, we first considered correct and error responses in the same model,

$$P{\left(correct\right)}_{t+1}={\left(1+e^{-(\beta_{0}+\beta_{1}*Acc_{t}+\beta_{2}*Pupil_{t}+\beta_{3}*(Acc_{t}*Pupil_{t}))}\right)}^{-1}$$ (3)

and the interaction effect was decomposed via simpler logistic regressions,

$$P{\left(correct\right)}_{t+1}={\left(1+e^{-(\beta_{0}+\beta_{1}*Pupil_{t})}\right)}^{-1}$$ (4)

fit to correct and error trials separately. We also fit versions of Eqs 3 and 4 that included an extra term to code for accuracy on *t*-1. Although the generally high collinearity between this regressor and accuracy on *t*+1 sometimes led to unstable parameter estimates and results from these fits are not reported here, the error-related pupil dilation/next-trial accuracy effects yielded by these models were nonetheless consistent with what we report in this manuscript (Fig 2d and 2f), suggesting that these effects are independent of previous-trial accuracy. In all cases in Eqs 1–4, group-level effects on the fitted *β* coefficients for individual predictors were tested via one-sample *t*-test (*H*_0_: *β* = 0). Variance inflation factors for all predictors across all models were <2.5, indicating weak multicollinearity.

We visualized the relationships between pupil dilation and next-trial behavior by pooling all trials of a given accuracy type across subjects, sorting by single-trial pupil dilation, grouping into 12 equal-sized bins, and calculating mean dilation and next-trial behavior per bin (Fig 2E and 2F). Both pupil dilation and log-transformed post-error RT were *z*-scored within-condition and within-subjects before pooling. In keeping with the approach of estimating PES independently from global performance fluctuations, we partialled out the effect of RT_t-1_ on RT_t+1_ via simple linear regression for the pooled-trial RT plots (Fig 2E).

## Results

Mean accuracy on the four-choice RT task was 87.1% (±SD = 4.4%). Mean RT was 619ms (±78ms) on correct trials and 631ms (±84ms) on error trials (*t*_19_ = -1.2, *p* = 0.2). When an error was committed, RTs on the following trial were slower than RTs on both pre-error trials (PES_robust_ = 30±46ms; *t*_19_ = 2.9, *p* = 0.009) and post-correct trials (PES_traditional_ = 22±38ms; *t*_19_ = 2.6, *p* = 0.018; Fig 1B). These complementary metrics of PES were highly correlated across individuals (*r*_20_ = 0.80, *p*<0.001) and did not differ reliably in magnitude (*p* = 0.2). Post-error accuracy (86.7±5.8%) did not differ from post-correct accuracy (87.3±4.5%; *p* = 0.6; Fig 1C), indicating that PES was not accompanied by a post-error improvement in accuracy at the trial-averaged level. We also examined whether the period preceding error commission was characterized by linear trends in RT and accuracy. RT grew progressively quicker over extended sequences of correct trials prior to error commission (*F*_1,19_ = 9.2, *p* = 0.007; Fig 1B). Moreover, a marginally significant trend existed toward progressively diminishing accuracy in the prelude to an error (*F*_1,19_ = 3.3, *p* = 0.08; Fig 1C).

Pupil size reliably increased following stimulus presentation. This dilatory response peaked approximately 300–800ms after response execution in the trial-averaged waveforms and was greater in magnitude on error compared to correct trials (*t*_19_ = 8.3, *p*<0.001; Fig 2A). Despite this clear effect of response accuracy, however, measurement of pupil dilation at the single-trial level revealed considerable overlap in the distributions of response amplitudes such that 81.8% of the distribution across error trials overlapped with the distribution across correct trials (Fig 2B). In contrast to the effect of response accuracy on evoked pupil dilation, unbaselined pupil diameter averaged over the one second prior to stimulus onset was not different for correct and error trials (correct = 3.23±0.08mm; error = 3.24±0.08mm; *t*_19_ = -1.0, *p* = 0.3).

We next determined whether the magnitude of the post-response pupil dilation on trial *t* predicted RT and accuracy on trial *t*+1. Within-subjects linear regression analyses incorporating both correct and error trials did not yield a reliable main effect of current-trial pupil dilation on next-trial RT (*β* = -0.02±0.02, *t*_19_ = -0.8, *p* = 0.4). However, this effect did interact with current-trial accuracy (*β* = 0.12±0.04, *t*_19_ = 2.7, *p* = 0.013). Post-hoc models to decompose this interaction revealed that the magnitude of the pupil dilation after an erroneous response positively predicted RT on the post-error trial (*t*_19_ = 2.3, *p* = 0.033), whereas this relationship was not present on correct trials (*t*_19_ = -0.7, *p* = 0.5; Fig 2C and 2E). Moreover, this relationship was independent of any effect of previous-trial RT (see Materials and Methods).

Lastly, within-subjects logistic regressions were used to assess the relationship between pupil dilation and accuracy on the following trial. As for the analysis of RT slowing, there was no main effect of current-trial pupil dilation on next-trial response accuracy in a model that considered both correct and error trials simultaneously (*β* = 0.03±0.04, *t*_19_ = 0.8, *p* = 0.4), although this effect was again found to interact with current-trial accuracy (*β* = 0.23±0.11, *t*_19_ = 2.2, *p* = 0.040). Follow-up regressions decomposing this interaction indicated that pupil dilation was positively related to next-trial accuracy on error trials (*t*_19_ = -2.9, *p* = 0.010), but this relationship did not extend to correct trials (*t*_19_ = -0.9, *p* = 0.4; Fig 2D and 2F).

## Discussion

In this study, we investigated the relationship between the error-related OR and post-error behavior on a choice RT task with extended intervals between consecutive trials. Previous studies that support the orienting account of PES have emphasized the negative consequences that orienting to an error might have on subsequent task performance [10,11]. On the contrary, we found that the magnitude of error-related pupil dilation, a common autonomic measure of physiological orienting, positively predicted both RT slowing and the likelihood of correct responding on post-error trials. Together, these features of behavior reflect an adaptive change of response strategy [4] rather than maladaptive OR-related interference. Thus our results suggest that the magnitude of the error-related OR is in fact a predictor of strategic post-error behavioral adjustment when the timing of the task facilitates resolution of the OR prior to subsequent trial onset. This conclusion can also be viewed as consistent with classical [19,20] and more recent [24,34] associations between phasic autonomic responses to novel or otherwise unexpected information and subsequent learning or adaptation: If participants generate predictions about their own task performance and these are violated by committing an error (e.g. [35,36]), one way of adapting and minimizing such prediction error in the future is to adjust the decision-making process to emphasize more cautious and accurate responding.

Although we presently observed a positive relationship between the error-related OR and post-error performance, it remains possible that the OR exerts an opposite, deleterious effect on subsequent performance when the interval between an error and the following trial is short [11,13,15,16], presumably because a still-developing error-related OR distracts from processing of a newly presented stimulus. Accordingly, an important next step to build on the present findings and further reconcile the orienting and adaptive control accounts of PES will be to demonstrate that a greater OR predicts increased PES and *decreased* post-error accuracy under short (<~800ms) RSIs. The pupil response is relatively slow and therefore ill-suited to addressing this question, though EEG signatures of orienting hold greater promise [12,13,37].

The PES observed on the current task was relatively small in magnitude, and there was no reliable post-error effect on accuracy at the trial-averaged level. Single-trial analysis of pupil dilation also indicated that errors that elicited small pupillary ORs were even followed by lower-than-average accuracy and relatively fast RTs, which are antithetical to an adaptive post-error adjustment of response strategy. Moreover, distributional analysis indicated that only a subset of error trials elicited pupillary ORs that were larger than those observed on correct trials. One plausible unifying explanation for this pattern of findings is that only a portion of errors on our choice RT task may have been explicitly detected. Error detection has previously been identified as a pre-requisite for elicitation of the error-related component of the autonomic OR [32,38] and, in some studies, was necessary for the manifestation of PES [32,39] and increased post-error accuracy [40]. Thus, while detected errors on our task would likely have elicited a large error-related OR and been followed by reliable post-error adjustment, undetected errors would not have elicited an error-related OR and may have been followed by a continuation of the linear trends of faster RTs and declining accuracy that were observed in the prelude to error commission. Additional support for the presence of undetected errors in our data is provided by the observation that average RT on error trials was numerically larger than RT on correct trials; this pattern is often observed when uncertainty exists over the correct response on a task, or when errors are due to attentional lapses and are less likely to be detected [38,41,42].

The physiology of the pupil dilatory response is quite complex: dilation can be determined by activation of the sympathetic division of the autonomic nervous system, inhibition of the parasympathetic division, or a combination of the two [43,44], and these influences are thought to be controlled both directly by nuclei in the brainstem and indirectly by a host of cortical and subcortical brain regions. As such, our findings afford limited insight into the neural structures that mediate the OR-related behavioural adjustments observed presently. Nonetheless, several lines of recent evidence implicate the brain’s neuromodulatory systems, and the locus coeruleus-noradrenergic (LC-NA) system in particular, as primary candidates for playing a dual role in regulating both pupillary dynamics and post-error behavior. Converging data indicate that pupil diameter provides a reliable proxy for fluctuations in LC activity in both monkeys [45,46] and humans [47]. Moreover, the LC and other neuromodulatory nuclei are transiently activated by unexpected and motivationally significant events [48,49] and are endowed with cortex-wide afferent projections that are well suited to coordinating the widespread neural adaptations [50,51] that follow error commission. Indeed, recent psychopharmacological [50], genetic [52] and brain stimulation [53] studies suggest that neuromodulatory systems exert a causal influence on PES magnitude. Further work is warranted to establish a mechanistic understanding of the role of these neural systems in the OR-linked post-error adjustments observed presently.
